# Evaluation of the Properties and Osteogenic Potential of a Novel Scaffold-Free Material, Spheroid Blocks Comprising Fused Spheroids of Human Periodontal Ligament Mesenchymal Stem Cells

**DOI:** 10.1155/sci/2681140

**Published:** 2025-10-14

**Authors:** Kotaro Sano, Satoru Onizuka, Takenori Suga, Yuichiro Oka, Sho Hironaka, Kohji Nakazawa, Hisataka Kondo, Kazunori Hamamura, Wataru Ariyoshi, Michihiko Usui

**Affiliations:** ^1^Division of Periodontology, Department of Regenerative Science in Conservative Dentistry, Kyushu Dental University, 2-6-1 Manazuru, Kokurakita-ku, Kitakyushu 803-8580, Fukuoka, Japan; ^2^Department of Life and Environment Engineering, The University of Kitakyushu, 1-1 Hibikino, Wakamatsu-ku, Kitakyushu 808-0135, Fukuoka, Japan; ^3^Department of Dental Hygiene, Aichi Gakuin University Junior College, 1-100 Kusumoto-cho, Chikusa-ku, Nagoya 464-8650, Aichi, Japan; ^4^Department of Pharmacology, School of Dentistry, Aichi Gakuin University, 1-100 Kusumoto-cho, Chikusa-ku, Nagoya 464-8650, Aichi, Japan; ^5^Division of Infections and Molecular Biology, Department of Health Promotion, Kyushu Dental University, 2-6-1 Manazuru, Kokurakita-ku, Kitakyushu 803-8580, Fukuoka, Japan

**Keywords:** cell block, osteogenesis, periodontal ligament mesenchymal stem cells, spheroid

## Abstract

Periodontal ligament stem cell spheroids are reportedly valuable for tissue regeneration; however, their application in vivo requires the use of a scaffold, which may raise safety concerns. We moulded human periodontal ligament mesenchymal stem cell (hPDLMSC) spheroids into blocks as a scaffold-free method for handling spheroids. We then examined the characteristics and osteogenic capabilities of hPDLMSC spheroid blocks in vitro and in vivo. First, the hPDLMSCs were seeded into microwell chips to form homogeneous spheroids, which were then seeded into net mould devices and cultured with rotary shaking to form hPDLMSC spheroid blocks. Next, real-time polymerase chain reaction (PCR) analysis, a live/dead assay and histological analysis were performed to investigate the properties of the hPDLMSC spheroid blocks. Finally, bone defects were created in mouse calvaria, and the defects were transplanted with hPDLMSC spheroid blocks; the osteogenic potential of the spheroid blocks was evaluated using three-dimensional (3D) micro-X-ray computed tomography (µCT) analysis and histological techniques. We identified that the expression levels of stemness markers and osteogenesis-related genes were higher in hPDLMSC spheroid blocks than in monolayer and spheroid-cultured hPDLMSCs. The live/dead assay and histological analysis revealed that there was almost no central necrosis in the hPDLMSC spheroid blocks, and hPDLMSC spheroid blocks formed nodules under osteogenic differentiation induction. Furthermore, the hPDLMSC spheroid block treatment group (without the use of scaffolds) exhibited both the nearly complete closure of the mouse calvarial bone defect and significantly increased bone microstructural parameters compared with the group in which hPDLMSC spheroids were transplanted in combination with scaffolds. Together, these findings indicate that hPDLMSC spheroid blocks possess excellent stemness and osteogenic potential, and may contribute to the establishment of novel scaffold-free therapies.

## 1. Introduction

Periodontitis is the most common infectious disease worldwide [1]. The pathogenesis of periodontal disease is a chronic inflammatory disease caused by periodontopathogenic bacteria. When bone resorption by activated osteoclasts exceeds bone formation by osteoblasts, it eventually leads to periodontal tissue destruction, including bone tissue (alveolar bone) [2]. Although various treatments, such as periodontal tissue regeneration therapy, have been devised and put into practical use [3, 4], it remains difficult to regenerate periodontal tissue completely. Enamel matrix derivatives and guided tissue regeneration are periodontal tissue regeneration therapies that act on undifferentiated mesenchymal stem cells (MSCs) derived from the periodontal ligament, which is reportedly an important structure for periodontal tissue regeneration [5]. MSCs can differentiate into multiple lineages and are therefore considered useful for tissue regeneration; they have been actively used in the field of tissue regeneration in recent years [6]. Human periodontal ligament (hPDL) MSCs (hPDLMSCs) can be isolated from the hPDL and are reported to have stemness and multi-differentiation potential, including osteogenic differentiation potential [7, 8].

A spheroid is a spherical mass formed by the three-dimensional (3D) aggregation of cells. The 3D arrangement of cells causes gradients in the access of each cell to oxygen and nutrients, and associated differences in proliferation speed. Thus, 3D cell arrangements have been reported to more closely duplicate an in vivo environment than monolayer cultures [9]. There are several methods for preparing spheroids, including the hanging drop culture [10], agitation or rotational culture [11] or using low-adherence well plates [12]; however, we focused on a method developed by Nakazawa et al that uses a microwell chip that can easily form and collect homogeneous spheroids [13, 14]. We have previously used this method to reveal that spheroids prepared from hPDLMSCs have superior stemness and osteogenic potential than two-dimensional (2D) cultures and exhibit osteogenic potential in vivo [15]. We have also demonstrated that hPDLMSC spheroids are effective for periodontal tissue regeneration [16].

In these previous studies, scaffolds were used for cell transplantation. The diameter of hPDLMSC spheroids is ~100 µm, meaning that scaffolds are generally necessary to assemble the cells into a 3D structure, as have been used in other spheroid transplantation experiments [17]. However, general concerns remain about the side effects of scaffolds, such as inflammatory reactions and allergies, because scaffolds are foreign to the body [18]. It has also been noted that scaffold use reduces the proportion of transplanted cells in the recipient and means that the cell environment in actual tissue cannot be mimicked [19, 20]. Furthermore, compared with a direct cell transplantation method, scaffolds are associated with issues in terms of the effort required for production and ease of use [21, 22]. To overcome the limitations of scaffold-based approaches, our group decided to apply the net mould method, which promotes the fusion and organisation of spheroids. In the net mould method—a type of tissue engineering technique—spheroids are added to the box-shaped cavities of net mould devices and cultured with shaking. Consequently, the spheroids contact each other and produce an extracellular matrix, thus promoting cell adhesion and tissue formation and resulting in the generation of 3D cell tissue. Other methods, such as 3D bioprinters and cell sheet engineering, have been established as scaffold-free technologies [23, 24], but the advantages of the net mould method include its simple operation and the ability to control the thickness of products. The use of 3D bioprinters requires large-scale, expensive, dedicated equipment; however, the net mould device is palm-sized and inexpensive, so making it easy to be implemented. In addition, as described above, the simple operation allows cell blocks to be obtained and contributes to the high reproducibility. In addition, our ultimate goal is to regenerate the periodontal tissues destroyed by periodontitis, and the net mould method, which makes it easy to control the size of the biomaterials according to the size of the periodontal tissue defect, may be preferable to the cell sheet engineering, which has a limited thickness of products [25]. Sakaguchi et al. [25] reported that they succeeded in constructing 3D tissue from human fibroblast spheroids without the use of scaffolds using a net mould. Cells with 3D structures can create 3D shapes by themselves and are highly safe because they do not require scaffolds for transplantation [26]. It therefore seems that applying a net mould to the field of tissue regeneration would be beneficial; however, there have been no reports of scaffold-free constructs created from periodontal ligament stem cell spheroids and no investigations of their characteristics and behaviour.

In periodontal tissue regeneration therapy, the remaining calculus and granulation tissue in the destroyed area (which are the cause of periodontitis) are surgically removed; regenerative materials are then placed into the alveolar bone defect. Periodontal tissue defects vary and may be complex. It has been reported that a combination of regenerative materials and scaffold-free graft materials, which can be densely filled into the defects, may be suitable for regenerating the alveolar bone and surrounding periodontal tissue, which will be key to preserving teeth [27].

We thus hypothesised that if we were able to create spheroid blocks from hPDLMSC spheroids using a net mould device, and if their characteristics had a positive effect on bone formation, they might contribute to the future development of periodontal tissue regeneration. We therefore aimed to form scaffold-free cell blocks from hPDLMSC spheroids and investigate their characteristics and functions in vitro and in vivo. Specifically, we first devised an experimental plan to confirm the state of internal necrosis in hPDLMSC spheroid blocks in vitro. We then evaluated gene expression related to stemness and osteogenic potential, and determined whether these spheroid blocks have osteogenic potential in bone defects in vivo.

## 2. Methods

### 2.1. Cell Isolation and Culture

As previously described [7], fresh hPDL tissue was harvested from the extracted wisdom teeth of systemically healthy patients who underwent extraction because of dental problems caused by their wisdom teeth; hPDLMSCs were immediately isolated. The data of the patients who cooperated in the sampling of hPDL are as follows: *n* = 15 (7 men and 8 women), mean age = 47.10 ± 21.51, probing pocket depth (PPD) = 2.54 ± 0.96 mm, target teeth were the bilateral mandibular wisdom teeth. Informed consent was obtained from all subjects. This study protocol was approved by the Kyushu Dental University Ethics Committee (Protocol #14-021), and the experimental procedures conformed to the Declaration of Helsinki and the guidelines of Kyushu Dental University. The extracted wisdom teeth were washed five times with phosphate-buffered saline (Thermo Fisher Scientific, Waltham, MA, USA) supplemented with antibiotics (100 U/mL penicillin and 100 µg/mL streptomycin; Wako Pure Chemicals, Osaka, Japan). The hPDL tissue was then carefully separated from the root surface to avoid contamination with other types of tissue and was cultured in digestion solution containing alpha minimal essential medium (Thermo Fisher Scientific) with collagenase type I (1 mg/mL; Wako) and dispase (1200 PU/mL; Wako) at 37°C for 60 min with continuous agitation. After enzymatic digestion, any debris in the cell suspensions was removed by filtration through a cell strainer (pore size, 70 µm; BD Falcon, Bedford, MA, USA). Next, the filtered cell suspensions were distributed into 100 mm tissue culture dishes (Iwaki, Shizuoka, Japan) and cultured in 10 mL of growth medium, consisting of alpha minimal essential medium containing 10% foetal bovine serum (Biosera, Nuaillé, France), 100 U/mL penicillin (Wako) and 100 µg/mL streptomycin (Wako). The medium was refreshed every 3 days. Colony-forming hPDLMSCs were selected and subcultured when they reached confluence. Initial experiments used hPDLMSCs that had been passaged fewer than five times. Our groups have already confirmed by flow cytometry analysis that hPDLMSCs isolated from the periodontal ligament using the same procedure express the MSC-positive markers CD29, CD44, CD73, CD90, CD105, CD106 and CD146 [15].

### 2.2. Spheroid Block Formation

Microwell chips tailored for spheroid formation were prepared as previously described [13]. The microwell chips, made of polymethyl methacrylate (PMMA) plates, had vertical and horizontal dimensions of 24 mm × 24 mm, with four microwells designed in the centre. The surface of each microwell (4 mm diameter, 1.5 mm depth) was coated with a platinum layer and then modified with 5 mM polyethylene glycol to prevent cell attachment, and the microwell chips were placed in a 35-mm culture dish (BD Falcon). A suspension of hPDLMSCs was then seeded at 3.0 × 10^5^ cells/well into the polyethylene glycol-coated microwells, and 2 mL of growth medium was added around the microwell chip. About 3 h after cell seeding, the dish was tilted and subsequently incubated with 5% CO_2_ at 37°C for 3 days, resulting in the formation of hPDLMSC spheroids. The number of seeded cells in the spheroid device was determined based on the report by Ariyoshi et al. [28], and the sizes of the formed spheroids were proportional to the cell numbers. hPDLMSC spheroids were observed using an all-in-one fluorescence microscope (BZ-9000; Keyence, Osaka, Japan) at 3 days after seeding.

The net mould devices used to form the 3D cell blocks have been described in detail previously [25]. The main components of the net mould (NM14-2, TissueByNet, Tokyo, Japan) are 12 stainless steel plates with a thickness of 100 µm. The bottom and top of the net mould each have a plate with 100-µm diameter stainless steel wires spaced 100 µm apart, and 10 plates with *φ* = 100 µm wires spaced 2, 4 and 6 mm apart are stacked between them. The net mould without the top net plate was placed in a 100 mm culture dish, and the cavity of the moistened net mould was filled with 40 hPDLMSC spheroids. The top net plate was then replaced, and the culture dish was filled with 15 mL of growth medium. The hPDLMSC spheroid fusions were cultured in the incubator at 37°C with 5% CO_2_ while being agitated at 45 rpm with a shaker (NA-M301, Nissinrika, Tokyo, Japan) to promote medium diffusion. The hPDLMSC spheroid fusions were cultured for 7 days, with medium changes every 2 days, to form hPDLMSC spheroid blocks.

### 2.3. Analysis of Spheroid Block Interiors

To evaluate the effects of blocking hPDLMSC spheroids on cell viability, a live/dead assay was performed using a Live/Dead Cell Staining Kit (Takara Bio, Shiga, Japan) according to the manufacturer's instructions. For this assay, we used hPDLMSC spheroids cultured in a microwell chip for 10 days or 7-day rotating-cultured hPDLMSC spheroid blocks that were generated from hPDLMSC spheroids cultured for 3 days in a microwell chip. The formation of a spheroid block requires the spheroid culture as a preliminary step. If each culture method was in the same period, only the spheroid block group would be cultured for a longer period, resulting in differences in the degree of cell development. Based on a report [29] conducted with this idea, we adopted this method of uniforming the overall period in order to compare and evaluate at the time of completion. Images were taken using a fluorescence microscope (BZ-9000; Keyence). The percentage of live cells, which were permeated with calcein and produced green fluorescence, and the percentage of dead cells, which were permeated with ethidium homodimer and produced red fluorescence, were quantified using ImageJ software (National Institutes of Health, Bethesda, MD, USA). We then attempted to histologically confirm the internal structure of the spheroid block. Forty hPDLMSC spheroids (3.0 × 10^5^ cells/spheroid) were cultured in a net mould device for 7 days before being fixed in 4% paraformaldehyde phosphate buffer (PFA) (Wako). Based on previous reports on histological staining of spheroids [30], cryosections were prepared, and the morphology and internal necrosis of the hPDLMSC spheroid blocks were examined using haematoxylin–eosin (H&E) and TdT-mediated dUTP nick end labelling (TUNEL) staining, respectively.

### 2.4. Quantitative Real-Time Polymerase Chain Reaction (PCR)

Real-time reverse-transcription PCR (RT-PCR) was performed as previously reported [16]. Total RNA was extracted from hPDLMSC spheroid blocks, hPDLMSC spheroids and monolayer cultures of hPDLMSCs on days 3 and 10 after seeding with ISOGEN II (Nippon Gene, Tokyo, Japan). Complementary DNA (cDNA) was generated by reverse transcription from RNA with a High-Capacity RNA-to-cDNATM Kit (Thermo Fisher Scientific) and amplified for 60 min at 37°C before being denatured for 5 min at 95°C. Quantitative real-time RT-PCR was performed in triplicate using FAST SYBR Green Master Mix (Applied Biosystems, Carlsbad, CA, USA) and the StepOnePlus Real-time PCR System (Applied Biosystems). The primer sequences used for PCR product detection are shown in Table 1. Relative gene expression was quantified using the comparative Ct method, and the total cDNA expression level of each sample was normalised using *GAPDH*-specific primers.

### 2.5. Osteogenesis Assay

The induction of osteogenic differentiation was performed in accordance with a previously published protocol [15, 16]. For monolayer cultures, 1.2 × 10^7^ hPDLMSCs were seeded onto a 100-mm culture dish; 1 day later, the culture medium was changed from growth medium to hMSC osteogenic induction medium (OIM)—containing dexamethasone, L-glutamine, ascorbate, penicillin/streptomycin, mesenchymal cell growth supplement, and b-glycerophosphate (Lonza, Basel, Switzerland)—and cultured for 7, 14 or 21 days. For the spheroid-cultured and spheroid block formation groups, hPDLMSCs were seeded onto 10 microwell chips at 3.0 × 10^5^ cells/well, and 1 day later, the culture medium in the microwell was replaced with OIM. In the spheroid-cultured groups, the culture was continued using microwell chips, whereas in the spheroid block formation groups, the formed hPDLMSC spheroids were placed into a net mould device after 3 days and cultured for up to 21 days. To genetically analyse each sample, RT-PCR was performed after 7, 14 and 21 days. In addition, histological staining was performed to visualise mineralisation inside the hPDLMSC spheroid blocks. Forty hPDLMSC spheroids (3.0 × 10^5^ cells/spheroid) induced to osteogenic differentiation by OIM were cultured in a net mould device for 21 days and fixed in 4% PFA. Cryosections were then prepared, and calcium deposits within the hPDLMSC spheroid blocks were revealed using von Kossa staining.

### 2.6. Transplantation of Spheroid Blocks Into Mouse Calvarial Defects

To evaluate the osteogenic capabilities of hPDLMSC spheroid blocks in vivo, we adopted a mouse calvarial model, which has a simpler bone morphology than periodontal tissue defects. The mouse calvarial defect model was created according to our previous report [15]. Thirty-six female C57BL/6N mice (aged 6 weeks; Kyudo, Saga, Japan) were used. The study protocols were approved by the Animal Care and Use Committee, Kyushu Dental University (Protocol #23-008) and were performed in accordance with Animal Research: Reporting In vivo Experiments (ARRIVE) guidelines and the National Institutes of Health guide for the care and use of laboratory animals. Mice were anesthetised by the intraperitoneal injection of a triple-mix anaesthetic consisting of medetomidine hydrochloride (0.15 mg/kg; Wako), midazolam (2 mg/kg; Dormicum, Astellas Pharma, Tokyo, Japan), and butorphanol tartrate (2.5 mg/kg; Vetorphale, Meiji Seika Pharma, Tokyo, Japan). The shaved scalp was then incised and elevated to allow access to the surgical site, the calvaria, at the top of the head. Next, a right-sided calvarial bone defect (3 mm diameter) was created using a trephine burr (Helmut Zepf, Seitingen-Oberflacht, Germany). Defects were (1) left untreated (sham operation); (2) filled with 40 spheroid-cultured hPDLMSCs (3.0 × 10^5^ cells × 40 = 1.2 × 10^7^ cells) combined with Matrigel (Corning Inc., Corning, NY, USA) as a carrier; or (3) filled with one hPDLMSC spheroid block (1.2 × 10^7^ cells). The 36 mice were divided into two groups (18 mice each) to be sacrificed 14 or 28 days after surgery; each group contained six mice from each of the three aforementioned groups. A previous study has demonstrated that Matrigel, which we used as a carrier for transplanting the hPDLMSC spheroids, does not have a significant effect on calvarial bone regeneration [15]. We therefore did not include a group in which only Matrigel was transplanted. After treating the bone defects, the scalp was returned to its original position and sutured with 7-0 silk sutures (Mani, Tochigi, Japan).

### 2.7. Micro-Computed Tomography Analysis

At 4 or 8 weeks after surgery, all mice were killed by anaesthetic overdose. Calvaria were then excised from the mice, and the healing of bone defects was radiologically analysed using 3D micro-X-ray computed tomography (µCT) and TRI/3D-BON (Ratoc Systems Engineering, Tokyo, Japan). Two bone microstructural parameters were selected based on previous studies [31]: bone volume fraction (BV/TV), which indicates the volume of trabecular bone obtained by the 3D analysis of bone density, and bone surface (BS), which indicates the 2D trabecular BS area. The same researcher measured BV/TV and BS within the circular bone defects of all mice; these data were then used for statistical analysis.

### 2.8. Histological Analysis

Histological analysis was performed according to methods previously established by our group [15]. After 3D µCT imaging, the calvaria (including the surgical site) was fixed with 4% PFA (Wako) for 2 days and decalcified with Morse's solution for 2 days at 4°C. Trimmed samples were then subjected to a series of dehydration steps, embedded in paraffin and cut into 4-µm sections. The sections were stained with H&E to identify morphological changes and were then observed using an all-in-one fluorescence microscope (BZ-9000; Keyence). The following parameters were measured in the H&E-stained images using ImageJ: (1) the percentage of defect closure/defect length, which was calculated by dividing the width of newly formed bone (i.e., the length of new bone that appeared inside the bone defect) by the width of the bone defect (i.e., the distance between the edges of the original bone defect); and (2) the rate of new bone formation/total defect area, which was calculated by dividing the area of newly formed bone by the area of the bone defect (i.e., the area enclosed by the cross section of the bone edges and the upper and lower parts of the virtual calvaria).

### 2.9. Statistical Analysis

Data are presented as the mean ± standard deviation, and all experiments were repeated three times. Statistical differences between groups were assessed using Student's *t*-test or Bonferroni correction. Differences with *p* < 0.05 were considered significant.

## 3. Results

### 3.1. Spheroid Block Formation

**Figure 1:**
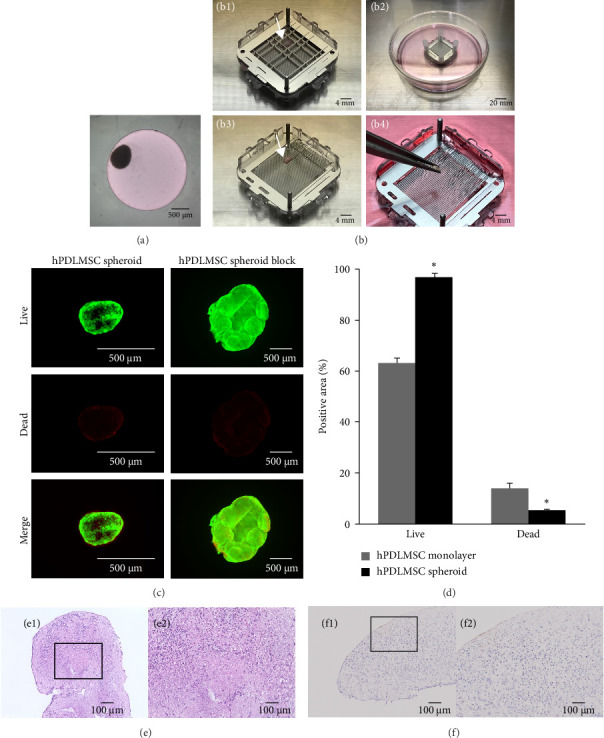
Characteristics of hPDLMSC spheroid blocks. (a) Microscopic image of an hPDLMSC spheroid. All spheroids were prepared at a density of 3.0 × 10^5^ cells/well using microwell chips at 3 days post-seeding. Scale bar = 500 µm. (b) Images of the process for preparing hPDLMSC spheroid blocks. (b-1) Forty hPDLMSC spheroids were placed into the cavity (arrowhead) of the net mould device. Scale bar = 4 mm. (b-2) A net mould device with an upper net plate was placed into a 100-mm culture dish with the growth medium, and the spheroids were cultured with rotary shaking. Scale bar = 20 mm. (b-3) hPDLMSC spheroids were fused 7 days later to become a single block (arrowhead). Scale bar = 4 mm. (b-4) It was possible to retain the generated hPDLMSC spheroid block using tweezers. Scale bar = 4 mm. (c) Staining of live and dead cells in hPDLMSC spheroids and hPDLMSC spheroid blocks. The hPDLMSC spheroids were observed 10 days after seeding on microwell chips, whereas the hPDLMSC spheroid blocks were observed 7 days after seeding on net moulds. Live cells were stained green with calcein, and dead cells were stained red with ethidium homodimer-1. Scale bars = 500 µm. (d) Percentage of live- and dead-cell-positive areas in the hPDLMSC spheroids and hPDLMSC spheroid blocks. Each stained area was measured using ImageJ. *⁣*^*∗*^*p* < 0.05 (compared with monolayer-cultured hPDLMSCs). To observe the internal states of the hPDLMSC spheroid blocks, frozen sections were prepared 7 days after seeding on the net mould before being subjected to haematoxylin–eosin staining (e) and TdT-mediated dUTP nick end labelling staining (f). (e-1, f-1) Images at lower magnification. Scale bars = 100 µm. (e-2, f-2) Higher-magnification images of the insides of the black frames in (e-1, f-1). Scale bars = 100 µm. hPDLMSC, human periodontal ligament mesenchymal stem cell.

**Figure 2:**
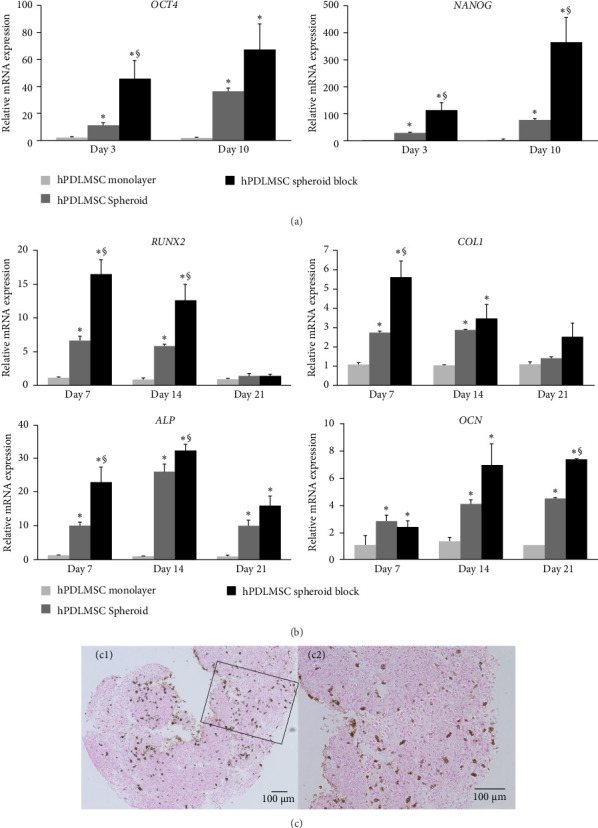
Enhancement of stemness and osteogenic differentiation in hPDLMSC spheroid blocks. (a) Expression of stemness markers in hPDLMSC spheroid blocks, hPDLMSC spheroids and hPDLMSCs grown in monolayer culture on days 3 and 10. The values were normalised to *GAPDH* expression. *⁣*^*∗*^*p* < 0.05 (compared with monolayer cultures of hPDLMSCs). *⁣*^§^*p* < 0.05 (compared with hPDLMSC spheroids). (b) Expression of osteogenesis-related genes in hPDLMSC spheroid blocks, hPDLMSC spheroids and monolayer hPDLMSCs cultured in OIM on days 7, 14 and 21. The values were normalised to *GAPDH* expression. *⁣*^*∗*^*p* < 0.05 (compared with monolayer-cultured hPDLMSCs). *⁣*^§^*p* < 0.05 (compared with hPDLMSC spheroids). (c) Images of sections of hPDLMSC spheroid blocks cultured in OIM for 21 days and stained with von Kossa stain. (c-1) Image at lower magnification. Scale bar = 100 µm. (c-2) Higher-magnification image of the inside of the black frame in (c-1). Scale bars = 100 µm. *ALP*, alkaline phosphatase; *COL1*, type 1 collagen; hPDLMSC, human periodontal ligament mesenchymal stem cell; *NANOG*, Nanog homeobox; *OCN*, osteocalcin; *OCT4*, octamer-binding transcription factor 4; OIM, osteogenic induction medium; *RUNX2*, runt-related transcription factor 2.

**Figure 3:**
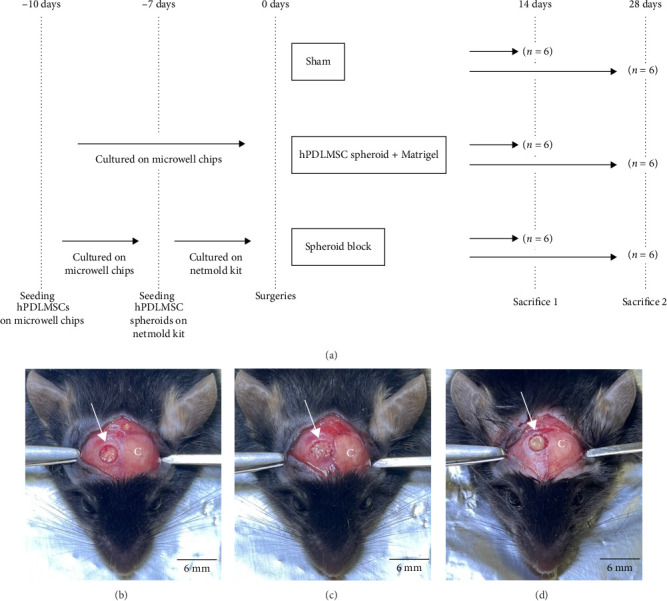
Surgical creation of mouse calvarial bone defects. (a) Study design of the transplantation assay. (b–d) Images of the surgical field focused around the mouse calvaria. A full-thickness flap was raised, and the calvaria was exposed. The surgical site was the mouse calvaria on the right side (white arrows). The bone defects were filled with 40 hPDLMSC spheroids with Matrigel (c), filled with one hPDLMSC spheroid block (d) or left untreated (sham group) (b). Scale bars = 6 mm. C, calvaria; hPDLMSC, human periodontal ligament mesenchymal stem cell.

**Figure 4:**
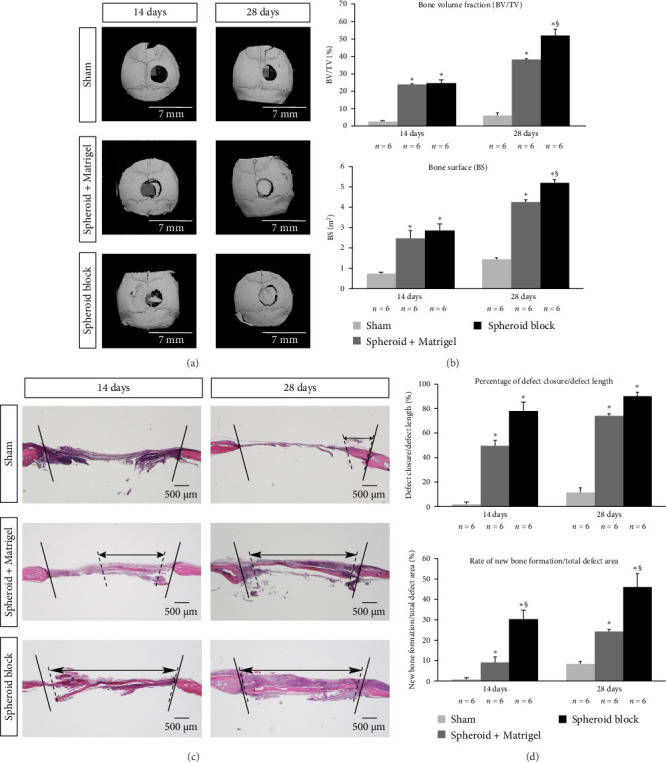
New bone formation after the transplantation of hPDLMSC spheroid blocks, analysed using 3D µCT and histological techniques. (a) 3D µCT images of calvaria around the surgical site. The bone defects were created on the right side of the calvaria using a 3-mm diameter trephine burr. Scale bars = 7 mm. (b) Quantification of new bone formation in horizontally sectioned µCT images. BV/TV and BS were evaluated using 3D bone morphometry software. *⁣*^*∗*^*p* < 0.05 (compared with sham). *⁣*^§^*p* < 0.05 (compared with hPDLMSC spheroids). (c) Haematoxylin–eosin-stained sections prepared on the coronal plane at the surgical sites of the mouse calvaria in each group. Solid lines indicate the edges of the bone defect; the area between the solid lines is the bone defect, created using a trephine burr. Dotted lines indicate the edges of the newly formed bone, and double-headed arrows indicate the width of the newly formed bone (as observed in the coronal plane). Scale bars = 500 µm. (d) Bone histomorphometric analysis of new bone formation in the untreated (sham), hPDLMSC spheroid-transplanted or hPDLMSC spheroid block-transplanted groups. The percentage of defect closure/defect length and rate of new bone formation/total defect area were calculated for each group. *⁣*^*∗*^*p* < 0.05 (compared with sham). *⁣*^§^*p* < 0.05 (compared with hPDLMSC spheroids). µCT, micro-X-ray computed tomography; 3D, three-dimensional; BS, bone surface; BV/TV, bone volume fraction; hPDLMSC, human periodontal ligament mesenchymal stem cell.

The microwell chip, which had different properties from those used in previous studies, allowed the hPDLMSCs to form spheroids. The diameter of the microwell was larger than that used in previous reports [15, 16]; however, the formed spheroids maintained their spherical shape (Figure 1a) and did not deform even when subjected to pressure from pipetting to remove them from the microwell chips. We attempted to fuse several hPDLMSC spheroids together to form a single block using a net mould device and demonstrated that this was feasible (Figure 1b-1–b-3). The hPDLMSC spheroid blocks generated 7 days after seeding onto the net mould were able to be handled with tweezers, suggesting that the use of a carrier was not necessary for in vivo experiments (Figure 1b-4). The average diameter of the hPDLMSC spheroids cultured in the microwell chip for 10 days was 1089 µm (*n* = 8), and the size of the hPDLMSC spheroid blocks prepared by culturing 40 hPDLMSC spheroids in the net mould for 7 days was 3.9 mm (length) × 2.9 mm (width) × 1.1 mm (height; *n* = 8).

### 3.2. Characterisation of hPDLMSC Spheroid Blocks

Central necrosis is a frequently reported problem with large spheroids [32, 33]. Because the spheroid blocks in the present study comprised an aggregation of many spheroids, we needed to determine whether the constituent cells were alive or dead. Although some dead cells were observed in the hPDLMSC spheroids, the cells were generally viable. In the hPDLMSC spheroid blocks, there were slightly more dead cells in the areas in which the spheroids overlapped; overall, however, live cells were predominant (Figure 1c,d). Furthermore, we stained cryosections of hPDLMSC spheroid blocks with H&E and TUNEL to examine the internal structure and apoptotic cells. H&E staining revealed no changes in nuclear morphology (i.e., nuclear atypia) characteristic of cancer, and the morphology of the nuclei constituting hPDLMSC spheroid blocks was considered normal (Figure 1e). Although TUNEL-positive cells were observed on the surface, few were found inside the spheroid blocks (Figure 1f).

Subsequently, we examined the expression of octamer-binding transcription factor 4 (*OCT4*) and Nanog homeobox (*NANOG*) using real-time RT-PCR to confirm the stemness of the hPDLMSC spheroid blocks. *OCT4* and *NANOG* are considered essential transcription factors for maintaining the pluripotency of stem cells [34]. On days 3 and 10 after seeding onto the microwell chips, the expression levels of both transcription factors were upregulated in the following order: monolayer culture, spheroids and blocks. For example, the expression levels of *OCT4* in hPDLMSC spheroid blocks were 26.7-fold higher (*p*=0.009) than monolayer culture and 4.19-fold higher (*p*=0.022) than spheroid culture on day 3. Similarly, the expression levels of *NANOG* in hPDLMSC spheroid blocks were 63.2-fold higher (*p*=0.008) than monolayer culture and 4.11-fold higher (*p*=0.09) than spheroid culture on day 3. In most cases, expression levels were significantly higher in blocks than in spheroids (Figure 2a). These results indicate that stemness is retained even after hPDLMSC spheroid fusion.

### 3.3. Osteogenic Properties of hPDLMSC Spheroid Blocks

To investigate whether osteogenesis-related genes are expressed in hPDLMSC spheroid blocks that have been induced to differentiate into bone using OIM, the expression levels of runt-related transcription factor 2 (*RUNX2*), type 1 collagen (*COL1*), alkaline phosphatase (*ALP*) and osteocalcin (*OCN*) were measured using real-time RT-PCR. After 7 days of culture, the expression levels of *RUNX2*, *COL1* and *ALP* mRNA were significantly upregulated in the hPDLMSC spheroid blocks compared with the spheroid cultures of hPDLMSCs (Figure 2b). In addition, the expression of *OCN* after 14 days of culture was higher in the hPDLMSC spheroid blocks than in the spheroid culture of hPDLMSCs (Figure 2b). Specifically, the gene expression on day 7 was 14.7-fold (*p*=0.004) for *RUNX2* and 5.17-fold (*p*=0.009) for *COL1* compared to the spheroid culture. Furthermore, the gene expression of *ALP* on day 14 was 39-fold (*p*=0.043) compared to the spheroid culture, and the gene expression of *OCN* on day 21 was 7.1-fold (*p*=0.017) compared to the spheroid culture. Together, these results suggest that hPDLMSC spheroid blocks have enhanced osteogenic potential via the upregulation of osteogenesis-related genes. We also stained prepared cryosections with von Kossa stain to investigate the osteogenic conditions inside hPDLMSC spheroid blocks cultured with OIM for 21 days. Calcified bone and calcium salt deposits were detected as black in the sections (Figure 2c). This finding similarly suggests that the hPDLMSC spheroid blocks have enhanced osteogenic potential.

### 3.4. Preparation of Mouse Calvarial Defects

Three types of treatments were performed on mouse calvarial defects to investigate the osteogenic potential of hPDLMSC spheroid blocks in vivo. The experimental groups and time schedules are shown in Figure 3a. Figure 3 shows images of the surgical sites after the creation of bone defects or the filling of the samples in each experimental group. In all groups, bone defects were surgically created in the right parietal region of the calvaria, as shown in Figure 3b. Although Matrigel (used as a scaffold) was required for the transplantation of 40 hPDLMSC spheroids (Figure 3c), the hPDLMSC spheroid block was able to be placed directly into the bone defect without a scaffold, and one block appeared to be an appropriate size to cover the surgical field (Figure 3d).

### 3.5. Analysis With 3D µCT

To facilitate a 3D understanding of post-operative bone morphology and obtain parameters related to bone status, 3D µCT was performed. We used BV/TV as a 3D trabecular structure parameter and BS as a 2D bone morphometry parameter. In the sham groups, where a circular hole was made in the calvaria with a trephine burr, almost no new bone formation was observed at 14 days post-surgery; even at 28 days post-surgery, there was little change (Figure 4a,b). By contrast, 3D µCT cross-sectional images of the harvested calvaria revealed that new bone had partially begun to form at 14 days after the transplantation of hPDLMSC spheroids or hPDLMSC spheroid blocks. Moreover, by 28 days after surgery, new bone formation was observed in a relatively large area in both treatment groups (Figure 4a). The 2D and 3D trabecular bone structure analysis demonstrated that new bone formation was significantly enhanced in the groups treated with hPDLMSC spheroids compared with the sham groups. Furthermore, in the hPDLMSC spheroid block-transplanted groups, new bone formation was significantly increased compared with the groups treated with hPDLMSC spheroids (Figure 4b). These data suggest that hPDLMSC spheroid block transplantation leads to improved formation of new bone compared with no treatment or spheroid-cultured hPDLMSC treatment.

### 3.6. Histological Evaluation of Mouse Calvarial Defects

To histologically evaluate the newly formed bone, coronal sections were stained with H&E. The endpoints were set in accordance with previous studies [15]. As expected, little new bone was observed in the sham groups at 14 and 28 days after surgery (Figure 4c,d). In the hPDLMSC spheroid-transplanted groups, thin bone appeared at the defect margin at 14 days after surgery, and the bone was wider and thicker by 28 days after surgery (Figure 4c). The defects filled with hPDLMSC spheroids were significantly more closed than those of the sham groups at both 14 and 28 days after surgery; however, the rate of new bone formation/total defect area was only 10%–30% because of the lack of thickness of the newly formed bone (Figure 4d). In the groups transplanted with hPDLMSC spheroid blocks, defect closure was further enhanced (although this effect was not significant compared with hPDLMSC spheroid transplantation). This was especially apparent at 28 days after surgery, when almost the full width of the defect was covered with new bone. Although the thickness of the new bone in the groups treated with hPDLMSC spheroid blocks did not recover to that of the host bone, the thickness was greater than that in the hPDLMSC spheroid-transplanted groups (Figure 4c,d). Moreover, the rate of new bone formation/total defect area was significantly higher in the groups treated with hPDLMSC spheroid blocks than in those treated with hPDLMSC spheroids (Figure 4d). Together, these data suggest that hPDLMSC spheroid blocks have excellent osteogenic potential in vivo.

## 4. Discussion

In 3D culture models, synthetic scaffolds have been widely used to promote cell proliferation and growth or to form tissue structures. However, synthetic scaffolds have different stabilities depending on the type, and there are concerns about the generation of byproducts because of decomposition, the impact on cell–cell interactions and other unexpected effects on cells [35]. In addition, the majority of the generated 3D cells are occupied by the scaffold, which may result in a relatively low cell density [19, 20]. Scaffold-free 3D culture models are therefore expected to be applied in regenerative medicine in the future.

Methods for constructing scaffold-free 3D culture models include the use of cell sheet engineering to construct 3D tissue and a 3D bioprinter that stacks cells on a base where fine needles are accumulated. Regarding the former, although a periodontal ligament cell sheet has been created and demonstrated good regenerative capabilities in animal experiments [23], its production requires the use of a temperature-responsive culture dish, which is difficult to operate. The latter refers to the novel bio 3D printer technology “Kenzan Method” developed by Nakayama et al. [24]. Using this method, cylindrical structures with the properties of cartilage [36] or cancellous bone [37] have been fabricated; however, the requirement for specialised, large-scale and accurate equipment in this technique is both a feature and a challenge.

Currently, clinical cases that may benefit from periodontal tissue regeneration therapy include deep and relatively narrow vertical bone defects and root furcation lesions [4, 38, 39]; their size is often less than 10 mm in length, width and depth, meaning that there is no need to create large tissues (such as organs). The net mould method used in the current study is a technique that does not require special equipment and can be used to easily and inexpensively construct 3D cellular tissues, especially compared with the other reported scaffold-free approaches for periodontal tissue regeneration. The maximum size of the cell block that can be created with our method is 14 mm in length and width, and we have determined that this volume is sufficient for its application to human periodontal tissue defects. Furthermore, we succeeded in creating cell blocks from multiple hPDLMSC spheroids; we then transplanted them into mouse calvarial defect models in a scaffold-free manner and confirmed new bone formation.

Incidentally, we attempted to measure the mechanical properties of the spheroid blocks in the present series of experiments. Based on the hardness test conducted in the past [40], we intended to conduct a hardness measurement test by using a constant speed to press the pressure surface of a Handy Durometer GS-754G (Teclock, Nagano, Japan), held in both hands vertically from directly above the sample, against a sample placed on a flat surface. The maximum value obtained within 1 s can then be defined as the hardness. However, our spheroid blocks were too soft to measure. Nonetheless, we noted no particular problems with operability; the spheroid blocks were able to be held with tweezers and transplanted into the calvarial defects. We therefore propose that this result indicates an advantage (that the material easily fits the complex morphology of bone) rather than a weakness in terms of the physical properties of our spheroid blocks.

A common problem in many tissue engineering studies is necrosis inside the tissue. When cells accumulate more densely to make the engineered tissue thicker and larger, the lack of vascular tissue becomes a greater obstacle. This issue also occurs in spheroids, which are aggregates of many cells; central necrosis is reportedly observed when diameters exceed 200 µm [32, 33]. The diameters of the hPDLMSC spheroids that were used to form hPDLMSC spheroid blocks were ~1000 µm; however, no clear central necrosis was observed, and dead cells seemed to instead be present on the spheroid surface. Ariyoshi et al. [28] used the same microwell chip as ours to create spheroids from ATDC5 cells (a cartilage cell line) and similarly reported a few dead cells scattered on the surface of the spheroids rather than in the centre. The occurrence of central necrosis in spheroids is considered to depend on various factors such as spheroid size, cell type and culture conditions [30, 41, 42]. One reason for our live/dead staining findings in hPDLMSC spheroids may therefore be the culture conditions in the microwell chip. Although hPDLMSC spheroid blocks were generated by the overlapping and fusing of many hPDLMSC spheroids, the numbers of dead cells appeared to be higher around the edges and overlapping areas, and the spheroids fused without losing their spherical structures. We thus hypothesise that the strong cohesive force of hPDLMSC spheroids may have prevented multiple spheroids from fusing together to form a single block with a smooth surface at the microscopic level. Although few studies have investigated spheroid fusion, the initial tack of two spheroids occurs rapidly within a few hours, suggesting the involvement of rotational movement by cytoskeletal contraction and myosin II [43].

Future challenges with this technique include further reducing the death of tissue-constituting cells and controlling the fusion of spheroids. One clue may come from a 2023 study that reported that the improved survival rate of MSC spheroids and their high anti-inflammatory effects upon transplantation are related to the downregulation of nucleotide-binding domain-like receptor protein 3 inflammasomes [44]. Nevertheless, future studies will need to provide deeper mechanistic insights into how spheroid fusion enhances stemness and osteogenic potential, possibly by discovering novel molecular pathways that are activated specifically in spheroid blocks. Our group does not intend to persist in scaling up the spheroid blocks. We plan to first focus on improving the survival rates of the spheroids themselves, and then apply this to the spheroid blocks. The reason for this is that, as mentioned above, in terms of the size alone, the current size of hPDLMSC spheroids is considered to be sufficient for clinical application of human periodontal tissue defects. Conventional microwell chips are made of PMMA, a material that is impermeable to oxygen and nutrients. By contrast, our collaborators, Nakazawa et al, have developed a microwell chip whose base is made of a polymer film with a honeycomb pattern and a regular porous structure. These authors have shown that the porous microwell base promotes the growth of HepG2 cell spheroids and maintains spheroid function better than PMMA microwell chips [45].

In addition, research is currently underway to introduce a vascular network inside the tissue to prevent cell necrosis. For example, when endothelial cell sheets are cultured, the endothelial cells reportedly infiltrate the underlying collagen gel with microchannels, thus successfully producing a thick cell sheet with a vascular network [46]. In 2023, a study was published that attempted to induce angiogenesis in spheroids by changing the period of mixing tumour cells with fibroblasts [47]. In this previous study, spheroids were formed in three different ways as follows: (1) tumour cells only, (2) tumour cells and fibroblasts at the same time and (3) tumour cells first and fibroblasts later, which were seeded onto an ultra-low attachment plate. The authors concluded that tumour spheroids created using the third (sequential) method had a relatively high density of fibroblasts surrounding the tumour spheroids, which may promote angiogenesis. Our group has previously reported a similar attempt [16], although we were focused on improving periodontal tissue regeneration ability and did not evaluate angiogenesis around the spheroids. However, we revealed that periodontal tissue regeneration was enhanced when vascular endothelial cells were co-cultured. It has also been reported that by placing two or more spheroids with the smooth muscle cells in the outer layer and the endothelial cells in the inner layer in close proximity, the spheroids fuse together to form a blood vessel-like structure with a larger diameter [48]. In the similar attempt to construct a vascular network, it was announced in 2024 that a new microvascular network was formed inside spheroids by co-culturing mouse adipose tissue-derived microvascular fragments and mouse osteoblastic cells MC3T3-E1 on a low-attachment plate [49]. These may be an important key for creating spheroid blocks with improved viability. In summary, we plan to first seed hPDLMSCs and then endothelial cells on a microwell chip with good nutrient permeability to create spheroids that will serve as the material for cell blocks, and then fuse them to create spheroid blocks with good viability and vascularisation.

In our in vivo experiments, new bone was formed by transplanting hPDLMSC spheroid blocks into mouse calvarial defects. When considering the regeneration pattern of new bone, our images from 3D µCT and H&E sections indicated that new bone did not heal from the defect margin. Many osteo-regenerative studies using calvarial defect models have been conducted and have reported that new bone is formed from the defect margin [50, 51]. The appearance of heterotopic ossification suggests that this regeneration pattern may be different from the normal bone repair mechanism. However, in some cases, newly formed bone was found in an independent location within the defect, as in our results. Lim et al. [52] implanted collagen sponges soaked with recombinant human bone morphogenetic protein 2 (rhBMP-2) and umbilical cord MSC-derived nanovesicles into mouse calvarial defects and reported that a high dose of rhBMP-2 (500 ng/mL) significantly promoted bone formation. The isolated new bone formation observed with 0.7 µg of rhBMP-2 was hypothesised to be caused by the collagen sponge acting as a scaffold for bone regeneration. In addition, Levi et al. [53] reported that human adipose-derived stromal cells used in combination with poly(lactide-co-glycolide) scaffolds caused the emergence of peninsular bone healing in mouse calvarial bone defects 2 weeks after surgery. This might be because the poly(lactide-co-glycolide) scaffold functions as a biomimetic, biodegradable scaffold into which human adipose-derived stromal cells can easily migrate. On the basis of these previous results, we hypothesised the following mechanism. We considered the possibility that the cell density of the hPDLMSC spheroid blocks was too low to promote regeneration of the host bone. The stem cells derived from hPDLMSC spheroid blocks migrated and remained within the defect, causing local differentiation and bone formation. Alternatively, bone inductive factors such as BMP (bone morphogenetic protein) or transforming growth factor (TGF)-β may not diffuse evenly within hPDLMSC spheroid blocks, and accumulate locally in the defects, resulting in bone formation only in that area. However, even by 28 days after surgery, we observed almost no integration with the host tissue. Although the size of the hPDLMSC spheroid block appeared appropriate for the bone defect, it may be that it was not large enough to sufficiently cover the defect and was therefore unable to act on the host tissue. Taking into account of this fact, a suitable microenvironment was formed only in the middle of the defects because it might be difficult for grafted cells to adhere and differentiate at the edge of the defects. Further investigation, such as immunohistochemistry to detect the localisation of hPDLMSCs, is required for this matter.

## 5. Conclusions

In the present study, hPDLMSC spheroid blocks exhibited stemness and osteogenesis in vitro and had low internal necrosis. Moreover, even without the use of a scaffold, the spheroid blocks had better osteogenic potential than hPDLMSC spheroids in vivo. Together, our findings indicate that hPDLMSC spheroid blocks can function without a scaffold and possess higher stemness and osteogenic potential than hPDLMSC spheroids. These data suggest that hPDLMSC spheroid blocks might have potential utility in regenerative medicine.

## Figures and Tables

**Table 1:** Primer sequences for real-time reverse-transcription polymerase chain reaction analyses.

Genes	Sequences of primers
*GAPDH*	F: 5′-GAAGGTGAAGGTCGGAGTC-3′
	R: 3′-GAAGATGGTGATGGGATTTC-5′

*OCT4*	F: 5′-AGCAAAACCCGGAGGAGT-3′
	R: 3′-CCACATCGGCCTGTGTATATC-5′

*NANOG*	F: 5′-TGAACCTCAGCTACAAACAG-3′
	R: 3′-TGGTGGTAGGAAGAGTAAAG-5′

*RUNX2*	F: 5′-AACCCTTAATTTGCACTGGGTCA-3′
	R: 3′-CAAATTCCAGCAATGTTTGTGCTAC-5′

*COL1*	F: 5′-AGGGCTCCAACGAGATCGAGATCCG-3′
	R: 3′-TACAGGAAGCAGACAGGGCCAACGTCG-5′

*ALP*	F: 5′-ACGTGGCTAAGAATGTCATC-3′
	R: 3′-CTGGTAGGCGATGTCCTTA-5′

*OCN*	F: 5′-GGTGCAGCCTTTGTGTCCAA-3′
	R: 3′-CCTGAAAGCCGATGTGGTCA-5′

Abbreviations: *ALP*, alkaline phosphatase; *COL1*, type 1 collagen; *GAPDH*, glyceraldehyde-3-phosphate dehydrogenase; *NANOG*, Nanog homeobox; *OCN*, osteocalcin; *OCT4*, octamer-binding transcription factor 4; *RUNX2*, runt-related transcription factor 2.

## Data Availability

The data that support the findings of this study are available from the corresponding author upon reasonable request.
